# The Face-Name Associative Memory Test as a Tool for Early Diagnosis of Alzheimer’s Disease

**DOI:** 10.3389/fpsyg.2018.01464

**Published:** 2018-08-14

**Authors:** José Rubiño, Pilar Andrés

**Affiliations:** Department of Psychology and Research Institute of Health Sciences (IUNICS), University of the Balearic Islands – Balearic Islands Health Research Institute (IdISBA), Palma de Mallorca, Spain

**Keywords:** Alzheimer’s disease, early diagnosis, mild cognitive impairment, neuropsychological evaluation, FNAME, associative memory

## Abstract

One current challenge for neuropsychologists is to design assessment methods capable of detecting cognitive deficits in the early or preclinical phases of Alzheimer’s disease (AD). The objective of this paper is to review the studies that have explored the use of the Face-Name Associative Memory Exam (FNAME) as a test for early diagnosis of AD. Studies looking at correlations between performance on the FNAME test and biomarkers in healthy people and studies comparing healthy controls and people with mild cognitive impairment are reviewed. These studies are based on the evidence that AD’s pathological process begins years before the most visible clinical manifestations. We conclude that the FNAME test may be a valuable tool for early diagnosis but that some important questions remain to be resolved in future research.

## Introduction

Aging involves brain and functional changes, and it is common to talk about a continuum from normal aging to Alzheimer’s disease (AD). Between the two ends of this continuum, subjective cognitive decline (SCD) refers to the perception of memory or other cognitive problems without impairment on standardized cognitive tests. Next comes mild cognitive impairment (MCI), considered an intermediate condition whereby changes in cognition exceed the normal, expected changes related to age, without affecting one person’s daily activities (Petersen et al., 1999). It constitutes a risk factor for progression to AD dementia (Jessen et al., 2014; Mitchell et al., 2014). According to the National Institute on Aging-Alzheimer’s Association’s diagnostic criteria (Albert et al., 2011), when these changes involve memory and there is positive evidence of certain biomarkers (e.g., beta-amyloid, tau, temporal atrophy, and/or frontoparietal hypometabolism), it is likely that the cognitive changes experienced by the person represent the initial symptoms of AD. This form of MCI is known as amnestic mild cognitive impairment (aMCI).

The prevalence of MCI in older people ranges between 14% and 18%, with aMCI being twice as prevalent as non-amnesic (naMCI) forms (Petersen et al., 2009). Existing data on conversion rates to AD vary among studies, with estimates ranging between 4% and 25% (Bozoky et al., 2000; Zaudig, 2002). The likelihood of conversion and its rapidity depends on the presence of biomarkers such as abnormal levels of beta-amyloid and tau proteins in the cerebrospinal fluid (CSF) (Petersen et al., 2010; García Ribas et al., 2014). Evidence of abnormalities in neuroimaging [atrophy of the medial temporal lobe in nuclear magnetic resonance (NMR) and temporoparietal hypometabolism in positron emission tomography (PET)] (Lippa and Chetelat, 2010) and the presence of the 𝜀4 allele (Livingston et al., 2017) also increase the likelihood of conversion to AD. Finally, it has been established that aMCI is more likely to constitute an early form of AD than naMCI (Petersen et al., 2009; Peraita et al., 2011).

AD is a neurodegenerative disease characterized by the presence of cognitive and behavioral impairments. Its onset is insidious and its course progressive, increasingly impairing daily life activities. AD is currently considered the most frequent cause of dementia in developed countries. Its exact etiology remains uncertain and is thought to involve multiple factors and complex causal mechanism, with age acting as the main risk factor (Morris and Becker, 2004). Since AD is a major public health problem with strong psychosocial and economic impacts, early diagnosis has become an important issue. Recent evidence has revealed that the physiopathological process underlying AD starts several years before the first clinical symptoms are observed (Price and Morris, 1999; Sperling et al., 2012), and that an early diagnosis and intervention would bring demonstrable economic benefits for healthcare systems (Barnett et al., 2014). If, for example, an effective early diagnosis method is introduced, the health care sector will bear the costs for diagnosis and treatment and its reimbursement but important benefits will be seen later in the course of AD in the social care sector (Wimo et al., 2014). Hence, achieving diagnosis in the preclinical or early phases of AD has become an important challenge.

Despite the value of biomarkers for the diagnosis of AD, the diagnostic of the illness remains “probable” and based on clinical symptoms (impairment of memory or other cognitive functions). In that sense, the neuropsychological evaluation plays a major role in early diagnosis. However, most available standardized neuropsychological tests are designed to detect cognitive deficits when at the AD stage and have limited value to uncover earlier cognitive changes. Recent work (e.g., Rentz et al., 2013; Loewenstein et al., 2017) has described the development of new neuropsychological tests, cognitively more demanding and sensitive, minimizing possible compensating strategies, and targeting specific vulnerabilities of people with early AD. One advantage of efficient neuropsychological tests would be to reduce the need for invasive examinations such as lumbar puncture for CSF biomarker analyses.

Episodic memory is typically affected in AD. The memory deficits typically presented by AD patients can be characterized as temporal, and sometimes they are known as the amnesic syndrome of the hippocampal type (Dubois and Albert, 2004), primarily identified by little improvement during recognition (Rémy et al., 2005) and by low delayed recall (Graham et al., 2004). In this paper, we review recent neuropsychological work on a type of memory that might be especially sensitive to age and cognitive decline and fit the requisites to be a useful test for early diagnosis: associative memory. This type of memory has proven to be especially sensitive to early AD (e.g., Parra et al., 2010; Della Sala et al., 2012), and the Face-Name Associative Memory Exam (FNAME) test (Rentz et al., 2011; Amariglio et al., 2012) has been suggested as a promising tool to measure it.

## The Face-Name Associative Memory Test

The FNAME is a cross-modal associative memory test initially developed by Rentz et al. (2011). Briefly summarized, it includes 16 face-name pairs and 16 face-occupation pairs, with a total of 32 pairs to remember. The administration procedure (Rentz et al., 2011; Amariglio et al., 2012) starts with the presentation of 16 faces (four faces per sheet), followed by the presentation of the same faces associated with 16 names (Face study phase). Participants are asked to look at each face for 2 s. The instructions mention that participants should read the name underneath the faces and try to learn each face-name pair. Immediately after the presentation of the pairs, the faces are shown one by one and participants are asked to recall the associated name. The correct number of pairs recalled is recorded as an initial learning score for the names. The same procedure is then repeated, this time pairing the faces with occupations instead of names. This phase is immediately followed by a recall phase in which participants are presented with the 16 faces, one by one, and asked to recall both the name and the occupation associated with each face. Finally, a delayed recall test is carried 30 min later, in which participants are asked again to recall name and occupation for each face.

One important characteristic of this test is its reliance on associative memory, which has been found to be especially sensitive to early stages of AD (Blackwell et al., 2004; Parra et al., 2010), particularly so when it involves cross-modal association as is the case with faces and names (Clare et al., 2002; Werheid and Clare, 2007). For example, using Functional Magnetic Resonance, Vannini et al. (2011) observed a deterioration of the neuronal activity during the memorization of face-name associations in people without clinical symptoms but with amyloid deposits (Sperling et al., 2009).

In the first published study using the FNAME, Rentz et al. (2011) used PET to measure the correlation between associative memory and the amyloid burden in cognitively healthy participants. The results showed that performance on the FNAME correlated with amyloid load in cortical regions of the brain related to memory systems such as the frontal cortex, posterior precuneus, posterior cingulate, and lateral parietal. The results also showed that this correlation was selective, since the selective reminding test (measuring episodic memory, SRT; Masur et al., 1989) did not reveal such correlation. More specifically, the correlation was found for the face-name associations and not for the face-occupation associations. Very similar results have been recently observed in SCD, with higher global amyloid deposition significantly related to worse performance on face-name associations but not with face-occupations or WMS-III Composite (Sanabria et al., 2018). Rentz et al. (2017) argued that face-name associations require greater episodic memory than face-occupations associations because the former involves the pairing of two pieces of information that are abstract and unique (Werheid and Clare, 2007). An example of the differential difficulty of names and occupations is the case of some names that represent an occupation (for example, “Baker”). Research has shown that it is easier to remember “Baker” when it is presented as a profession than when it is presented as a proper name (James et al., 2008; Amariglio et al., 2012). Finally, Rentz et al. (2017) results show that the FNAME does not produce ceiling effects as other memory tests do, thereby making it potentially useful to detect subtle changes in preclinical phases of AD.

While relatively few studies have used the FNAME so far, some normative and psychometric data are available. For example, Amariglio et al. (2012) reported normative data from a sample of cognitively healthy American adults aged 58–90, showing a decline of performance with age and superior performance for face-occupation associations relative to face-name pairs. Test–retest reliability was high, as was the convergent validity of the FNAME components with another measure of episodic memory (SRT).

Recently, Papp et al. (2014) have developed a modified version of the task (FNAME 12), with fewer stimuli and more learning trials. In this version, memory is tested by a recognition test in which participants are presented with pairs of faces and pick that which they had learned, and pick the associated name/occupation from a list of possible options. Compared to healthy controls, patients with MCI showed significantly lower scores. Psychometric analyses showed that this test exhibited excellent convergent validity with the original FNAME-16 test and with other measures of episodic memory such as the FCSRT (Grober and Buschke, 1987). The data also showed that the performance on FNAME-12 improved with higher educational levels. However, they did not show a significant relationship with age, probably due to the reduced age ranges and the sample size included in the study.

The FNAME was recently adapted for the Spanish-speaking in the United-States (S-FNAME, Quiroz et al., 2014), and its psychometric and demographic characteristics assessed in a Spanish sample by Alegret et al. (2015b) and compared to those of the Wechsler Memory Scale (WMS-III; Wechsler, 1997). The results confirm the superior performance for face-occupation compared to face-name associations, and a correlation between performance in S-FNAME and WMS-III. Contrary to what was observed in Paap et al.’s study, age and gender had a significant effect on recall performance, but educational level did not.

Later, Alegret et al. (2015a) examined the ability of S-FNAME to discriminate between cognitively healthy and MCI individuals aged 49 or over. S-FNAME discriminated poorly between the groups (sensitivity = 52.9), contradicting the results of Paap et al.’s study (which used FNAME-12). However, as Alegret et al. (2015a) argued, this might have reflected the fact that the participants in their MCI group were volunteers with memory complaints instead of participants selected based on their performance in neuropsychological tests. The results also showed that performance on S-FNAME did not correlate with depressive symptoms or subjective memory deterioration.

The Face-Name Test is currently being used in some ongoing studies. For example, Morris et al. (2012) have included it in the “Dominantly Inherited Alzheimer’s Network” to develop an extensive prospective study of biomarkers in individuals at risk of autosomal dominant AD (ADAD). In this context, it is used to determine the chronopathology of the clinical, cognitive, neuroimaging, and fluid biomarkers of AD. It is also being used in investigations with anti-amyloid treatments in asymptomatic AD for the secondary prevention of AD (Sperling et al., 2012). The results from these studies remain to be published. Finally, a recent study has shown changes in Face-Name recall performance following hormonal changes in women, with premenopausal and perimenopausal women outperforming postmenopausal women (Rentz et al., 2017).

Therefore, although the rationale behind the use of the FNAME test and the existing results suggest that it may be a relevant tool in clinical neuropsychology, we observe some contradictory results among studies, and fundamental questions remain open that should be resolved in new studies. For example, to what extent is the relationship with and age and education strong? Can the test really discriminate between cognitively healthy people and people with MCI who come to consultation with cognitive complaints?

Finally, it is also worth mentioning that FNAME is a suitable task to be computerized. Compared to pen-and-paper tests, computerized tests reduce the risk of administration and scoring errors and ensures a more standardized procedure.

## Conclusion

Considering aging as a continuum from normal to pathological functioning, neuropsychologists must design assessment tools with reliable and sensitive instruments for the early detection of cognitive deficits that may constitute early stages of AD. A priority is to design neuropsychological tests with good sensitivity and specificity (see Weissberger et al., 2017), which correlate with the typical AD lesions, and thus improve the probability of early detection and diagnosis, thereby increasing the chances of early interventions to help maintain good quality of life and well-being for patients for as long as possible. Such outcome would prolong the autonomy and independence of our elders in their daily social, family, and work environment.

The ability to remember faces and names causes frequent complaints in older adults. In that sense, the FNAME affords more ecological validity (Zelinski and Gilewski, 1988; Leirer et al., 1990) than numerous other neuropsychological tests and is well suited to respond to the requirements of a good neuropsychological test. The use of this test has been suggested as a useful and sensitive test to detect early deficits in AD following the pivotal study by Rentz et al. (2011). These authors showed crucial inverse correlations between performance on the face-name associations section and amyloid deposition in frontal and posterior cortical regions in still healthy people. In contrast, neither face-occupation associative memory nor he SRT were significantly related to amyloid deposition. Similar results have been observed in SCD (Sanabria et al., 2018). In that context, the F-NAME is a promising test that shows promise for early diagnosis of AD. However, contradictory results have also been reported and future research should endeavor to address the issue of the test’s ability to detect differences between healthy and MCI individuals.

Sensitivity and specificity, two fundamental parameters to assess the goodness of a diagnostic test also remain to be studied. Other questions such as the effect of age or education should also be experimentally explored, and future studies should address these issues using adequate versions of the test in different countries.

## Author Contributions

JR and PA wrote the manuscript and approved it in its final form.

## Conflict of Interest Statement

The authors declare that the research was conducted in the absence of any commercial or financial relationships that could be construed as a potential conflict of interest.
